# Optimization of Culture Media for Human Umbilical Cord-Derived Mesenchymal Stem Cell Production

**DOI:** 10.1155/sci/4806605

**Published:** 2025-11-14

**Authors:** Wanglong Chu, Muyun Liu, Yan Shangguan, Fangtao He, Xiuping Zeng, Tao Guo, Tongjing Li, Fen Zhang, Qingfang Wang, Jianfu Wu, Zhenzhong Zhong, Xiao Liang

**Affiliations:** ^1^Center for Cell Preparation, Shenzhen Beike Biotechnology Co., Ltd., Shenzhen, China; ^2^R&D Center, National Engineering Research Center of Foundational Technologies for CGT Industry, Shenzhen, China; ^3^R&D Center, Shenzhen Kenuo Medical Laboratory, Shenzhen, China

**Keywords:** α-MEM, culture media, manufacturing, MSCs, serum-free

## Abstract

The development of robust and scalable culture systems is essential for the clinical-scale production of human umbilical cord (UC)-derived mesenchymal stem/stromal cells (MSCs) (UC-MSCs). While various basal and serum-free media are commercially available, systematic comparisons of their efficacy in supporting the expansion and functional properties of UC-MSCs remain limited. In this study, we conducted a comprehensive evaluation of multiple culture systems, including basal media (α-MEM, DMEM, and DMEM/F12) supplemented with human platelet lysate (HPL), and commercial serum-free media (Corning MSC Xeno-Free SFM, NutriStem XF Medium, Prime-XV MSC Expansion XSFM), for their ability to sustain UC-MSCs proliferation, maintain phenotypic properties, and support functional potency. The results demonstrated that all basal media supported cell growth, with α-MEM (Gibco) and DMEM/F12 showing superior performance over DMEM. Among serum-free formulations, Prime-XV with 2% HPL yielded the highest primary culture output and the shortest population doubling (PD) time (PDT) during passaging. Notably, cells expanded in commercial serum-free media exhibited reduced diameter and higher uniformity. Functional analyses revealed that NutriStem XF Medium supplemented with 2% HPL elicited the strongest immunomodulatory effects in mixed lymphocyte reactions (MLRs). Furthermore, all media maintained trilineage differentiation capacity and satisfied International Society for Cellular Therapy (ISCT) phenotypic criteria. Critically, no tumorigenic potential was detected in vitro or in vivo. Large-scale manufacturing using the selected medium (NutriStem XF + 2% HPL) confirmed consistent expansion kinetics, high viability, stable marker expression, and functional potency across seven production batches. This study provides a rigorous and clinically relevant framework for selecting culture media that ensure both scalability and functional integrity of UC-MSCs, highlighting the promise of serum-free systems for therapeutic manufacturing.

## 1. Introduction

Human mesenchymal stem/stromal cells (MSCs) (hMSCs) are of significant value in clinical applications and are abundant in neonatal and adult tissues. Following extraction and in vitro expansion, these materials have therapeutic potential for a wide range of diseases, including neurological disorders, joint issues, cardiovascular diseases, graft-versus-host disease (GVHD), pulmonary conditions, inflammatory bowel disease (IBD), liver diseases, and diabetes. The therapeutic efficacy of MSCs primarily stems from their abilities in tissue regeneration and immune modulation, with paracrine effects playing a crucial role [1, 2, 3, 4]. Despite more than 50 years since Friedenstein's pioneering studies in the 1960s, the functional diversity and heterogeneity of MSCs underscore the absence of a single marker for their identification. The International Society for Cellular Therapy (ISCT) has outlined minimal criteria to define MSCs, including their ability to adhere to plastic in culture; high expression of CD105, CD73, and CD90 (≥95%); and lack of expression of CD45, CD34, CD14 or CD11b, CD79a or CD19, and HLA class II (≤2%). Furthermore, MSCs can differentiate into osteoblasts, adipocytes, and chondroblasts in vitro [5].

Bone marrow, adipose tissue, and the umbilical cord (UC) are the three most commonly sourced types of MSCs used in clinical trials, and the safety and efficacy of MSC administration have been confirmed [6, 7]. Among these, human UC-derived MSCs (UC-MSCs) have garnered increased attention owing to their low ethical concerns, strong proliferative capacity, and ease of procurement, making them particularly promising for advancing medical treatments and therapies [8, 9, 10, 11].

Typically, MSCs must be cultured in vitro to obtain large numbers of cells for therapeutic use. The isolation and expansion methods of UC-MSCs have been extensively studied, and good manufacturing practice (GMP)-compliant manufacturing processes have been established [12, 13]. Among the factors influencing MSC culture, the cell culture medium plays a crucial role in producing MSCs, providing the necessary environment for cell growth and survival. This medium includes glucose, amino acids, lipids, trace elements, and growth factors that are essential for promoting MSC proliferation while maintaining their morphology, phenotype, and multilineage differentiation potential. Therefore, the selection of cell culture medium significantly impacts the safety and efficacy of MSCs, necessitating rigorous screening during process establishment [14, 15].

Traditional hMSC culture systems often utilize basic media such as αMEM or DMEM supplemented with fetal bovine serum (FBS). The basic medium supplies fundamental components for cell growth, while FBS contributes growth factors, hormones, and adhesion factors that are critical for cell adhesion and proliferation. Despite its widespread use, FBS has drawbacks, including the potential risks of unknown virus transmission due to its animal blood origin and immunological rejection as a heterologous component. Moreover, significant batch variations in the FBS can lead to process instability [16, 17]. Human platelet lysate (HPL) has emerged as an alternative to FBS, addressing concerns related to heterologous materials, although potential batch-to-batch differences still exist [18]. Currently, basic culture media supplemented with FBS or HPL are widely employed in scientific research and clinical applications.

In response to the limitations of these culture systems, serum-free, defined culture systems tailored for MSCs have been developed by numerous cell therapy companies and suppliers, and have already entered commercial use [19, 20]. These specialized MSC culture media have been shown to support MSC proliferation and maintain basic characteristics. However, the choice of the culture medium for hMSCs remains a challenge for most research institutions and manufacturing enterprises. Some studies have compared the characteristics of MSCs cultured in different culture systems [20, 21], but there is still a lack of in-depth comparisons of the media used for MSC production. This study, for the first time, illustrated the process of culture media selection, primarily investigating platelet lysates and serum-free culture media. It systematically compares basic culture media and commercially available media, evaluates different serum-free options, examines tumorigenicity, and assesses the stability of large-scale multibatch production. These efforts aimed to provide valuable insights into detailed methods for selecting appropriate cultural media.

## 2. Materials and Methods

### 2.1. Isolation of UC-MSCs

UC samples were obtained from eight parturient donors aged 20–35 years via cesarean section, and written informed consent was obtained. Three donors were used for basal media screening, an additional three donors for commercial media comparisons, and two donors for scale-up production. The collection process was approved by the Medical Ethics Committee of Shenzhen Baoan District Maternity and Child Healthcare Hospital (Shenzhen, China) following the Declaration of Helsinki. UC-MSCs were isolated using enzymatic digestion methods, as previously described [22]. Briefly, UCs were decontaminated, segmented, and opened to extract Wharton's jelly. Wharton's jelly was minced into 1–4 mm^3^ fragments and digested with collagenase NB6 GMP (Nordmark Biochemicals, Germany) at a concentration of 0.4 PZ U/mL at 37°C for 3 h. Following digestion, the cell mixture was diluted, passed through a 100 μm cell strainer, centrifuged to remove the supernatant, and then seeded into a 25 cm^2^ flask or a cell factory (Nunc, Thermo Fisher Scientific, USA). The first medium exchange occurred after 5 days, with subsequent exchanges every 3 days until the cell confluence reached 60%–80%. These MSCs were harvested using recombinant trypsin (CTS TrypLE Select, Gibco, USA) and designated P0. The cells were continuously passaged at a density of 4500–5500 cells/cm^2^ in culture media at 37°C with 5.0% CO_2_ and harvested when the cell confluence reached 85%–95%.

### 2.2. Selection and Evaluation of the Medium for UC-MSC Culture

To identify the optimal basal culture medium for UC-MSC expansion, α-MEM (Lonza, USA), α-MEM, DMEM, and DMEM/F12 (Gibco, USA) were used. These media were supplemented with 5% or 10% HPL (Stemulate, Sexton Biotechnologies, USA or PLTGold, Mill Creek Life Sciences, USA) to create complete culture media. UC-MSCs derived from three independent donors at passage 2 were plated in 25 cm^2^ flasks with each medium and cultured until 85%–95% confluency (typically achieved within 3 days) for harvesting. The population doubling time (PD) (PDT) was compared to identify the culture system with the best proliferative capacity.

Subsequently, three commercial serum-free MSC culture media were compared: Corning MSC Xeno-Free SFM (Corning, USA), NutriStem XF Medium (Biological Industries, Israel), and PRIME-XV MSC Expansion XSFM (FUJIFILM Irvine Scientific, USA). Unlike basal media, these formulations typically contain defined growth factors (e.g., epidermal growth factor [EGF], bFGF, platelet-derived growth factor [PDGF]-bb). Based on manufacturer recommendations and prior studies [23, 24], supplementation with 2% or lower HPL was sufficient to initiate cell adhesion and proliferation in commercial media; in contrast, basal media require 5% or more HPL supplementation under standard protocols [25]. To test the proliferative capacity of these serum-free media, three different methods were used: media only, media supplemented with vitronectin (Gibco, USA) in a 5 µg/25 cm^2^ flask, and media supplemented with 2% HPL. Three UC samples were digested and plated using different culture methods for primary culture. After primary culture, the harvested cells were passaged to passage 4 (P4) using the same culture system as the previous generation, with a consistent passaging density and culture conditions. P4 cells were evaluated for cell morphology, proliferative capacity, multilineage differentiation potential, cell surface marker expression, colony-forming unit-fibroblast (CFU-F) assay, and lymphocyte proliferation inhibition rate. The optimal culture system was selected based on a comprehensive evaluation.

### 2.3. Cell Morphology, Counting, Viability, and Proliferation Analysis

Cell morphology and confluence were observed using an inverted microscope (IX73; Olympus, Japan). Cell counting, viability, and diameter (μm) were analyzed using an automatic cell counter (Vi-Cell Blu, Beckman Coulter, Inc., USA) using the trypan blue exclusion method. Cell proliferation capacity was compared by calculating the PDT using the formula (*X* = *T* × log_2_/(log*N−*log*X*_0_), where (*T*) is the time between initial plating and harvest, (*N*) is the total number of harvested cells, and (*X*_0_) is the initial number of cells plated. PDs were calculated using the formula (*X* = (log*N*–log*X*_0_)/log_2_).

### 2.4. UC-MSC Immunophenotyping and Multilineage Differentiation Assays

MSC surface antigen expression was analyzed by flow cytometry (FACSCalibur, BD Biosciences, USA) with CellQuest Pro software. The cells were stained with antibodies against human CD73-PE, CD45-FITC, CD34-PE, CD14-FITC, CD79a-APC, HLA-DR-PerCP, CD146-PE (BD Biosciences, USA), CD105-APC (Thermo Fisher Scientific, USA), CD90-FITC, and SSEA-4-APC (BioLegend, Inc., USA), which were used as isotype controls.

The multilineage differentiation potential of MSCs was tested using OriCell osteogenic, adipogenic, and chondrogenic differentiation kits (Cyagen Biosciences Inc., China), according to the manufacturer's instructions. For osteogenic and adipogenic differentiation, the cells were cultured in a 6-well plate until they reached 70%–100% confluence, after which the medium was replaced with differentiation medium, and the medium was changed every 3 days. After 2–4 weeks, the cells were stained with Alizarin Red S or Oil Red O solution to assess differentiation. For chondrogenic differentiation, 300,000–400,000 cells were cultured in differentiation medium for 3–4 weeks and stained with Alcian Blue. The stained sections were analyzed under an inverted microscope (100 × magnification; CKX53, Olympus, Japan). Cells grown in regular medium served as negative controls.

### 2.5. CFU-F Assay

To assess clonogenic potential and stemness preservation under each culture condition, a CFU-F assay was conducted. MSCs were plated in triplicate at a density of 100 cells/cm^2^ in 6-well dishes. After 14 days of culture, cells were fixed with methanol for 30 min and stained with 0.1% crystal violet (Sigma) for 45 min. The dishes were washed twice with distilled water, air-dried, and colonies containing more than 50 cells were scored.

### 2.6. Mixed Lymphocyte Reaction (MLR) Assay

The immunomodulatory ability of MSCs was evaluated using a MLR assay by coculturing MSCs with CFSE (Solarbio, China)-labeled peripheral blood mononuclear cells (PBMCs). MSCs were plated in a 12-well plate, and CFSE-labeled PBMCs were stimulated with 10 ng/mL PHA (Beyotime Biotechnology, China) and cocultured at a 1:5 MSC-to-PBMC ratio for 4–5 days in RPMI-1640 medium (Gibco, USA) supplemented with 10% FBS. After incubation, lymphocyte proliferation was determined by monitoring the reduction in CFSE fluorescence using a BD FACSCalibur flow cytometer. PBMC cultures without MSCs and PHA stimulation served as negative controls, whereas the positive controls were treated with PHA. The suppression rate (%) was calculated as (1 − (positive control − sample)/(positive control) × 100%).

### 2.7. Tumorigenicity Analysis

Maintaining genetic stability and tumorigenicity during long-term passage is crucial in evaluating media, including soft agar assays, cell tumorigenicity (animal tests), and karyotype analysis. MSCs at passages P2, P5, and P10 were tested.

The soft agar colony formation assay was performed in a 6-well plate precoated with a base agar layer (1% soft agar solution in DMEM) (Takara, Japan) for 30 min at room temperature. First, 1 × 10^4^ MSCs were suspended in DMEM and 0.7% agar solution and plated onto precoated plates. HeLa cells were used as a positive control. The plates were incubated at 37°C with 5% CO_2_ for 2–3 weeks, fixed with 4% paraformaldehyde (Feike Biotechnology, China), and stained with 0.005% crystal violet (Solarbio, China). Colony formation was observed by bright-field microscopy.

To verify whether MSCs can spontaneously transform and form tumors in vivo, 4- to 7-week-old female BALB/c nude mice were subcutaneously injected with 1 × 10^7^ cells. The experimental groups (*n* = 10/group) were defined by cell type. As a positive control, mice received 1 × 10^7^ HeLa cells, which exhibit tumor-initiating capability. Normal human cells served as a negative control. Animals were observed for more than 16 weeks, twice per week for the first 3–6 weeks, and then weekly. A valid test revealed no tumor growth in the negative control group and progressive tumor growth in at least nine animals in the positive control group. At the end of the observation period, all the animals, including the control animals, were killed, and the proliferation of inoculated cells at the injection site and other sites (such as the heart, lung, liver, spleen, kidney, brain, and local lymph nodes) was observed by eye and microscope. These tissues were fixed with 4.0% formaldehyde solution, sliced, and stained with hematoxylin and eosin, and a histopathological examination was performed to determine whether the inoculated cells formed tumors or had metastasized. Animal suffering was minimized according to the Guide for the Care and Use of Laboratory Animals.

Karyotyping was performed using the G-banding technique. Cell division was halted in metaphase using 0.3 μg/mL colchicine (Solarbio, China) at 37°C for 2–3 h. After washing and trypsinization, the cells were suspended in 0.075 M KCl hypotonic solution and incubated at 37°C for 30–40 min. The cells were fixed in methanol and glacial acetic acid (3:1), dropped onto clean slides, and dried at 70°C for 3 h. Slides were then treated with trypsin, rinsed with saline, and stained with a 1:20 dilution of Giemsa solution. The band quality was evaluated under a microscope (100× magnification). Mitoses were captured using specialized software, with a minimum of 20 metaphases analyzed per sample.

### 2.8. UC-MSC Proliferation in Large-Scale Manufacturing

To verify the effect of the medium on the proliferation capacity and stability of MSCs, large-scale manufacturing was performed from passages 2–5. P2 cells were thawed and seeded into a 2-layer cell factory, harvested at 85%-95% confluence, and then seeded into five 5-layer cell factories to generate P4 cells. The P4 cells were then seeded into 16 10-layer cell factories, harvested at 85%–95% confluence to obtain P5 cells, and finally harvested. Various parameters, including cell number, viability, proliferation fold, PD, PDT, markers expression, multilineage differentiation potential, and lymphocyte suppression rate, were analyzed across seven batches from two donors.

### 2.9. Statistical Analysis

Statistical analyses were performed using GraphPad Prism, version 9.5.0. Correlations were assessed using simple linear regression analysis. Paired *t*-tests were used for comparisons between two groups, while one-way ANOVA was used for comparisons among multiple groups. Quantitative data are reported as the mean ± standard deviation (SD). A *p*-value less than 0.05 was considered to indicate statistical significance.

## 3. Results

### 3.1. Evaluation of Basal Medium for UC-MSCs Culture

The performance of P2 UC-MSCs cultured in four basal media, namely, α-MEM (Lonza), α-MEM (Gibco), DMEM (Gibco), and DMEM/F12 (Gibco), was assessed. Because of the inability of basic media to support MSC culture independently, supplementation with 5% or 10% HPL was implemented based on previous studies. The results demonstrated successful cell proliferation across all basal media formulations irrespective of the presence of 5% or 10% HPL.

**Figure 1:**
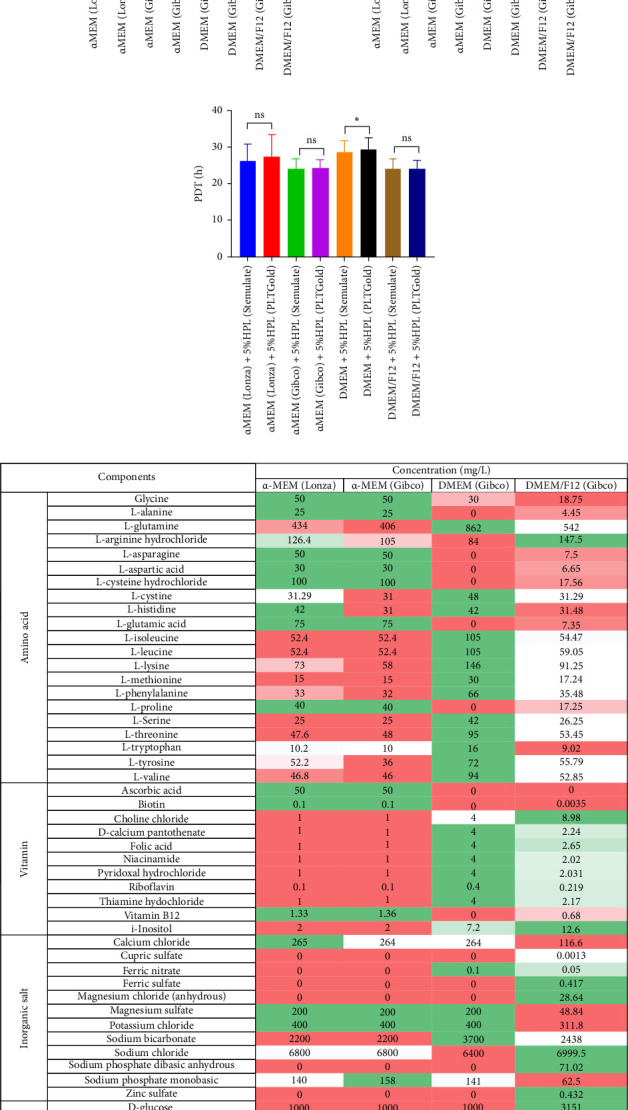
Evaluation of basal media for UC-MSCs culture. (A) Population doubling time (PDT) of P2 UC-MSCs cultured in different basal media supplemented with 5% or 10% human platelet lysate (HPL). (B) Cell diameter measurements of UC-MSCs cultured in the respective basal media. (C) Comparison of the PDT performances of HPLs from Stemulate and PLTGold in different basal media. (D) Comparative analysis of the composition and content of basal media from different suppliers. *⁣*^*∗*^*p* < 0.05, *⁣*^*∗∗*^*p* < 0.01, *⁣*^*∗∗∗*^*p* < 0.001. The data are presented as the mean± SDs (*n*=3).

Analysis of the PDT revealed differences between the basal media. Specifically, cells cultured in α-MEM (Gibco) with 5% or 10% HPL exhibited a slightly lower PDT than those cultured in α-MEM (Lonza) at similar HPL concentrations, although the difference was not statistically significant (*p* > 0.05). Furthermore, α-MEM (Gibco) and DMEM/F12 displayed comparable PDT values, which were lower than those observed in DMEM, indicating a greater proliferation rate in the former two media. Notably, DMEM supplemented with 5% HPL showed a significantly greater PDT than α-MEM (Gibco) or DMEM/F12 supplemented with the same HPL concentration (*p* < 0.05) (Figure 1A). The cell diameter measurements agreed with the PDT results, with DMEM exhibiting the largest cell diameter among the basal media tested (*p* < 0.05) (Figure 1B). Interestingly, the proliferative capacity of HPLs from Stemulate and PLTGold was similar, except in DMEM, where a significant difference was noted between the two brands (*p* < 0.05) (Figure 1C).

Furthermore, a comparison of the composition and content of the basal media from the suppliers revealed variations. Basic media include amino acids, vitamins, inorganic salts, ribonucleosides, deoxyribonucleosides, and other components. Variances between α-MEM (Lonza) and α-MEM (Gibco) were primarily observed in the contents of ribonucleosides and deoxyribonucleosides. DMEM exhibited a simpler ingredient profile than the other media, while α-MEM (Gibco) and DMEM/F12 differed in the species and quantity of amino acids, vitamins, inorganic salts, and other components, including ribonucleosides and deoxyribonucleosides.

Ultimately, α-MEM (Gibco), DMEM/F12, and HPL (Stemulate) were selected for further research based on their ability to support UC-MSC culture.

### 3.2. Comparison of Different Media for UC-MSCs Initial Culture and Passage Culture

To further investigate the impact of different culture media on MSC proliferation and quality, we conducted a comparative evaluation of several commercial serum-free MSC culture media—Corning MSC Xeno-Free SFM, MSC NutriStem XF Medium, and Prime-XV MSC Expansion XSFM—along with α-MEM (Gibco) and DMEM/F12—through both primary isolation and subsequent passages.

**Figure 2:**
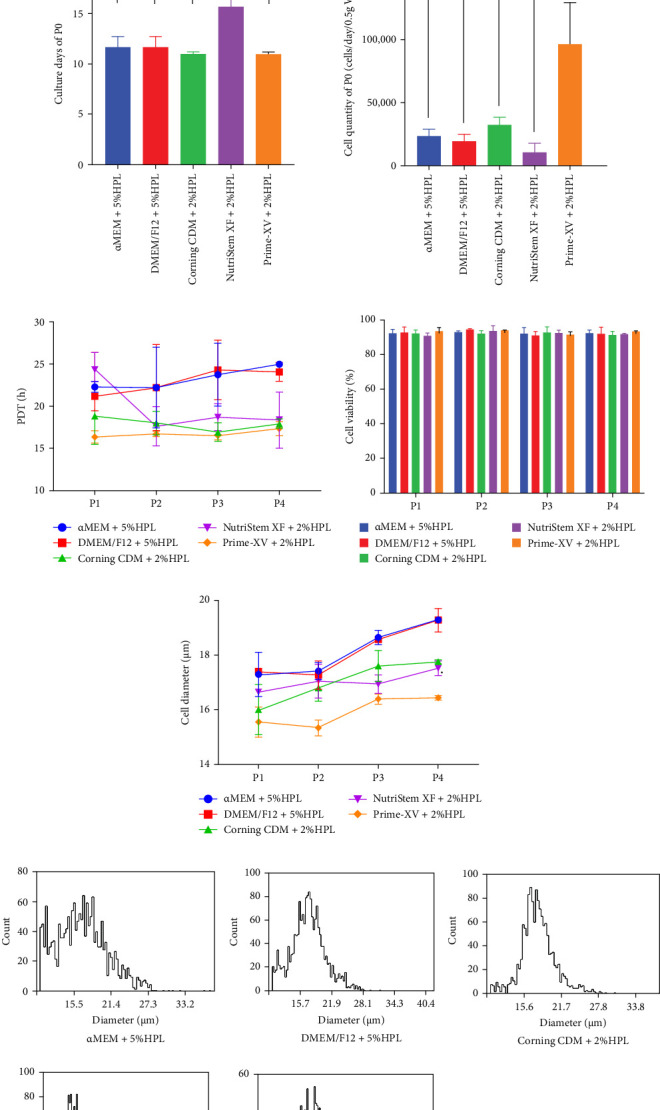
Comparison of different media for UC-MSCs initial and passage culture. (A) Proliferation of UC-MSCs in serum-free MSC culture media during primary culture (P0), comparing media only, vitronectin coating, and supplementation with 2% HPL. (B) The cell yield and culture days of UC-MSCs from 0.5 g of Wharton's jelly in various media during primary culture: α-MEM with 5% HPL, DMEM/F12 with 5% HPL, Corning MSC Xeno-Free SFM with 2% HPL, MSC NutriStem XF Medium with 2% HPL, and Prime-XV MSC Expansion XSFM with 2% HPL. (C) Population doubling time (PDT) of UC-MSCs from P1 to P4 in different culture media. (D) The viability of UC-MSCs cultured in different media from P1 to P4 did not significantly differ and exceeded 90%. (E) Average cell diameter from P1 to P4 in different media. (F) Cell diameter distribution of P3 UC-MSCs cultured in different media. (G) Representative morphological characteristics of UC-MSCs cultured in different media. Bar: 500 µm. *⁣*^*∗*^*p* < 0.05, *⁣*^*∗∗*^*p* < 0.01, *⁣*^*∗∗∗*^*p* < 0.001. The data are presented as the mean± SDs (*n*=3).

Initially, none of the serum-free MSC culture media supported MSC proliferation during primary culture, except for Prime-XV MSC Expansion XSFM, which was coated with vitronectin (Figure 2A). However, supplementation with 2% HPL promoted proliferation in all three serum-free MSC culture media.

The initial culture durations to reach 60%–80% confluence were 11.64 ± 1.09 days for both α-MEM and DMEM/F12 supplemented with 5% HPL, 10.97 ± 0.21 days for Corning MSC Xeno-Free SFM supplemented with 2% HPL and Prime-XV MSC Expansion XSFM supplemented with 2% HPL, and 15.66 ± 1.32 days for MSC NutriStem XF Medium supplemented with 2% HPL, with MSC NutriStem XF Medium requiring a longer culture duration (*p* < 0.01). The P0 yield from 0.5 g of Wharton's jelly was as follows: 2.32 × 10^4^ ± 5.61 × 10^3^ cells/day for α-MEM with 5% HPL, 1.90 × 10^4^ ± 5.85 × 10^3^ cells/day for DMEM/F12 with 5% HPL, 3.20 × 10^4^ ± 6.48 × 10^3^ cells/day for Corning MSC Xeno-Free SFM with 2% HPL, 1.01 × 10^4^ ± 7.39 × 10^3^ cells/day for MSC NutriStem XF Medium with 2% HPL, and 9.64 × 10^4^ ± 3.34 × 10^4^ cells/day for Prime-XV MSC Expansion XSFM with 2% HPL. Notably, Prime-XV MSC Expansion XSFM with 2% HPL yielded significantly more cells than the other media (*p* < 0.01). (Figure 2B).

Next, P0 cells were passaged to P4 in the respective culture systems as described in the Materials and Methods section. Commercial serum-free MSC media show a clear advantage, with cultured cells exhibiting a lower PDT. The average PDT from P1 to P4 was 23.31 ± 2.20 h for α-MEM with 5% HPL, 22.94 ± 2.40 h for DMEM/F12 with 5% HPL, 17.92 ± 1.36 h for Corning MSC Xeno-Free SFM with 2% HPL, 19.75 ± 3.15 h for MSC NutriStem XF Medium with 2% HPL, and 16.72 ± 0.54 h for Prime-XV MSC Expansion XSFM with 2% HPL. Although MSC NutriStem XF Medium with 2% HPL had a longer doubling time in the P1 generation, the cells subsequently entered a rapid expansion phase (Figure 2C). The cell viability exceeded 90% across all media, with no significant differences observed (Figure 2D). The cell diameter increased gradually from P1 to P4 in all culture media: 17.29 ± 0.58 to 19.30 ± 0.05 μm for α-MEM with 5% HPL, 17.38 ± 0.09 to 19.29 ± 0.31 μm for DMEM/F12 with 5% HPL, 16.01 ± 0.65 to 17.76 ± 0.05 μm for Corning MSC Xeno-Free SFM with 2% HPL, 16.65 ± 0.46 to 17.52 ± 0.19 μm for MSC NutriStem XF Medium with 2% HPL, and 15.55 ± 0.39 to 16.44 ± 0.06 μm for Prime-XV MSC Expansion XSFM with 2% HPL. Compared with α-MEM or DMEM/F12, commercial MSC serum-free media resulted in smaller cell diameters (Figure 2E). Furthermore, the cell diameter distribution showed that cells in the Corning MSC Xeno-Free SFM and Prime-XV MSC Expansion XSFM groups were more uniform, with fewer small-diameter cells and large-diameter cells, especially compared to those in the α-MEM group (Figure 2F). All cells exhibited spindle-shaped morphology; however, cells cultured in commercial MSC serum-free media appeared more three-dimensional and less flat. Prime-XV MSC Expansion XSFM-cultured cells displayed a notably elongated and slender morphology (Figure 2G).

### 3.3. Quality Analysis of UC-MSCs Cultivated in Different Media

**Figure 3:**
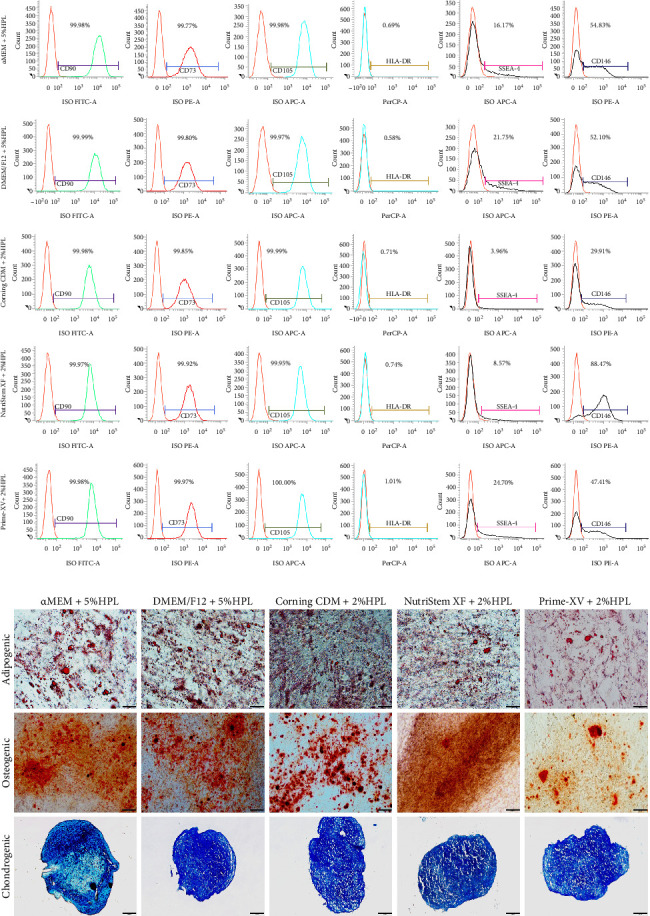
Analysis of marker expression and differentiation potential in UC-MSCs cultured in various media. (A) Representative images showing the expression levels of CD90, CD73, CD105, HLA-DR, SSEA-4, and CD146 in UC-MSCs cultured in α-MEM supplemented with 5% HPL, DMEM/F12 supplemented with 5% HPL, Corning MSC Xeno-Free SFM supplemented with 2% HPL, MSC NutriStem XF Medium supplemented with 2% HPL, and Prime-XV MSC Expansion XSFM supplemented with 2% HPL. (B) Representative images showing the differentiation of UC-MSCs into osteogenic, adipogenic, and chondrogenic lineages in different media. Bar: 100 µm.

A comprehensive quality analysis was subsequently conducted to evaluate the impact of different media on UC-MSCs. This analysis included the examination of marker expression, differentiation potential into specific lineages, and assessment of biological activity. Through this analysis, insights into the phenotypic and functional characteristics of UC-MSCs cultured in different media were obtained. All cultured MSCs exhibited robust expression levels of the CD73, CD90, and CD105 markers, exceeding 95%. Moreover, the expression of HLA-DR markers was consistently below 2%, meeting the minimal criteria defined by ISCT. Notably, the data showed variations in SSEA-4 and CD146 expression across different media, suggesting distinct stemness and functionalities (Figure 3A).

**Figure 4:**
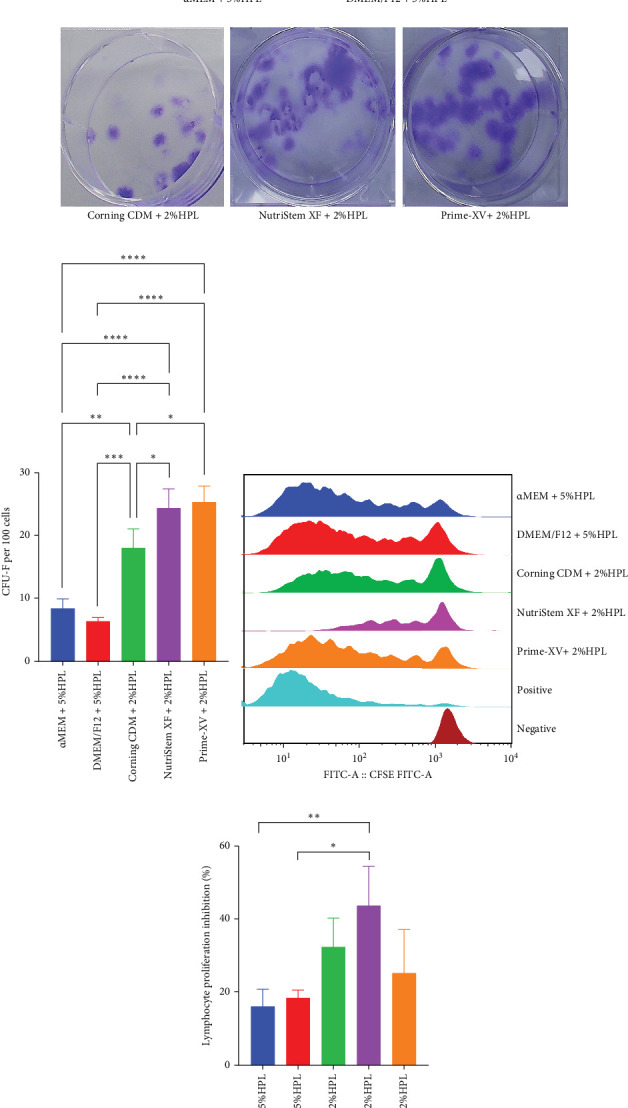
Analysis of CFU-F and immunomodulatory ability in UC-MSCs cultured in various media. (A) A representative photograph of the CFU-F of UC-MSCs cultured in different media. (B) The average number of colonies of UC-MSCs cultured in different media. (C) A representative example of the immunomodulatory capacity of UC-MSCs cultured in different media in a mixed lymphocyte reaction assay. (D) The average immunomodulatory capacity of UC-MSCs cultured in different media, depicted as the percentage suppression of lymphocyte proliferation. *⁣*^*∗*^*p* < 0.05, *⁣*^*∗∗*^*p* < 0.01, *⁣*^*∗∗∗*^*p* < 0.001, *⁣*^*∗∗∗∗*^*p* < 0.0001. The data are presented as the mean± SDs (*n*=3).

In addition to their phenotypic characteristics, differentiation potential, and clonogenic potential, the immunomodulatory ability is crucial for MSC therapy in vitro. MSCs cultured in all media were capable of differentiating into osteogenic, adipogenic, and chondrogenic lineages (Figure 3B). CFU-F assays revealed that NutriStem XF + 2% HPL and Prime-XV + 2% HPL supported significantly higher colony numbers (24.33 ± 3.06 and 25.33 ± 2.52 colonies/100 cells, respectively) versus basal media (α-MEM + 5% HPL: 8.33 ± 1.53; DMEM/F12 + 5% HPL: 6.33 ± 0.58; *p* < 0.0001) (Figure 4A, B). MSCs cultured in NutriStem XF Medium supplemented with 2% HPL exhibited the greatest decrease in lymphocyte proliferation in the MLR assay. The mean suppression rates were 16.02% ± 4.68% for α-MEM supplemented with 5% HPL, 18.38% ± 2.13% for DMEM supplemented with 5% HPL, 32.29% ± 8.01% for Corning MSC Xeno-Free SFM supplemented with 2% HPL, 43.70% ± 10.76% for MSC NutriStem XF Medium supplemented with 2% HPL, and 25.15% ± 11.97% for Prime-XV MSC Expansion XSFM supplemented with 2% HPL (Figure 4C, D).

A comprehensive comparison of the media is summarized in Supporting Information 1: Table S1. Based on these results, NutriStem XF Medium with 2% HPL was selected for subsequent experiments because it demonstrated higher proliferative capacity during passaging, the greatest immunomodulatory ability, and superior regional availability, despite Prime-XV exhibiting the fastest proliferation.

### 3.4. Tumorigenicity of MSCs Cultivated in Serum-Free Medium

**Figure 5:**
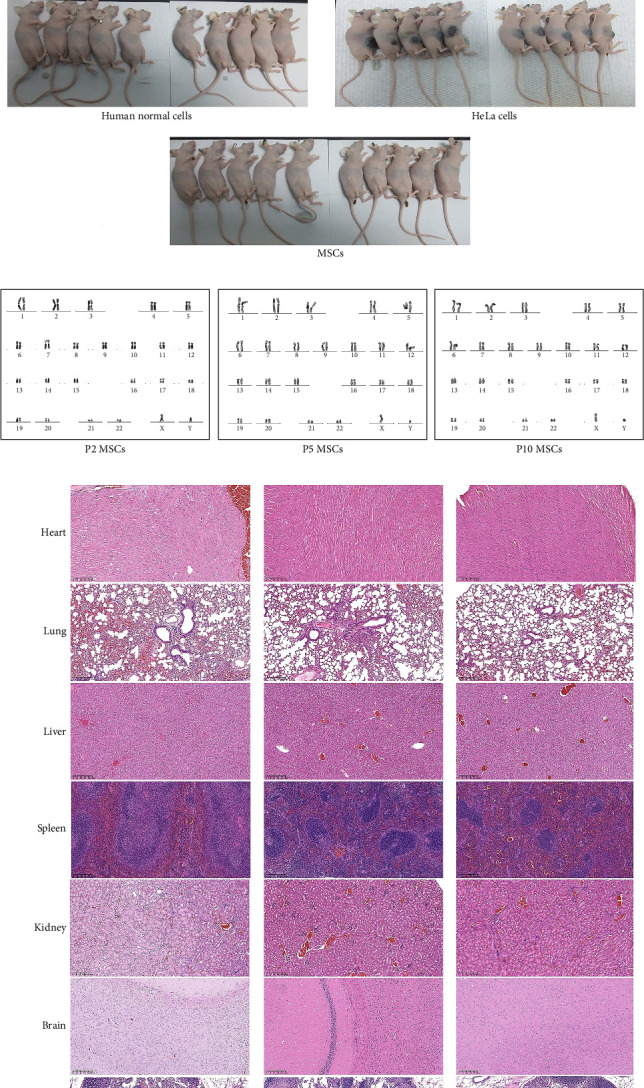
In vitro and in vivo tumorigenicity assessment of MSCs cultured in NutriStem XF medium supplemented with 2% HPL. (A) Soft agar colony formation assay results showing a lack of colony formation in MSCs at passages P2, P5, and P10, in contrast to HeLa cells, which formed colonies as expected. Bar: 200 µm. (B) In vivo tumorigenicity assessment through subcutaneous inoculation of MSCs into nude mice. No progressive tumor nodules were observed at the injection sites up to 112 days postinoculation in the MSC and normal human cell groups, demonstrating the nontumorigenic potential of MSCs under these conditions. In contrast, tumors were identified at the injection sites in the group transplanted with HeLa cells. (C) Histopathological examination of the heart, lung, liver, spleen, kidney, brain, lymph nodes, and injection site in nude mice. No evidence of tumor formation was found in the MSCs group. In the positive control group injected with HeLa cells, tumors were observed at the injection sites, whereas no tumors were detected in the negative control group injected with normal human cells. Bar: 200 µm. (D) Karyotype analysis of MSCs at passages P2, P5, and P10. Normal karyotypes without any numerical or structural chromosome abnormalities were observed.

To assess the safety profile of MSCs cultivated in a serum-free medium, a comprehensive tumorigenicity assay was conducted both in vitro and in vivo. This study focused on evaluating the potential tumorigenic properties of MSCs cultured in NutriStem XF Medium supplemented with 2% HPL at passages P2, P5, and P10, which represent different stages of cell bank generation and clinical application. In the in vitro soft agar colony formation assay, none of the MSC generations (P2, P5, and P10) exhibited colony formation, in contrast to HeLa cells, which formed colonies as expected (Figure 5A). This observation indicated the nontumorigenic nature of the MSCs under the culture conditions.

Subsequent in vivo assessment involved subcutaneous inoculation of MSCs into nude mice to evaluate the tumor formation potential. Nodule formation at the injection site was closely monitored, and bidirectional measurements were taken regularly to track nodule progression, stability, or regression. The absence of progressive nodules, particularly those showing regression, was indicative of nonneoplastic behavior. Tumor formation was considered positive if at least two out of 10 animals developed progressive tumors at the injection or metastatic sites, with histopathological analysis confirming the similarity of the cellular morphology between the formed tumors and the inoculated cells. Additional analysis was required if only one tumor developed to confirm tumorigenicity.

Remarkably, the subcutaneous inoculation of MSCs into nude mice revealed no progressive tumor nodules at the injection sites in any of the 10 female nude mice, even up to 112 days postinoculation (Figure 5B). Histopathological examination further supported these findings, showing no evidence of tumor formation (Figure 5C). Notably, the positive control mice exhibited tumor formation, while the negative control mice did not, validating the experimental setup.

Moreover, karyotype analysis of MSCs at passages P2, P5, and P9 revealed normal karyotypes without any numerical or structural chromosome abnormalities (Figure 5D), confirming the genetic stability of the MSCs under serum-free culture conditions.

### 3.5. Selection of Media for Large-Scale Production of UC-MSCs

To evaluate the suitability of the selected culture system for clinical and industrial applications, we conducted a large-scale manufacturing study spanning seven independent production batches derived from two donor sources. Cells were expanded from P2 through P5 using a scaled-up process involving multilayer cell factories (2-layer to 10-layer systems), simulating commercial production settings.

**Figure 6:**
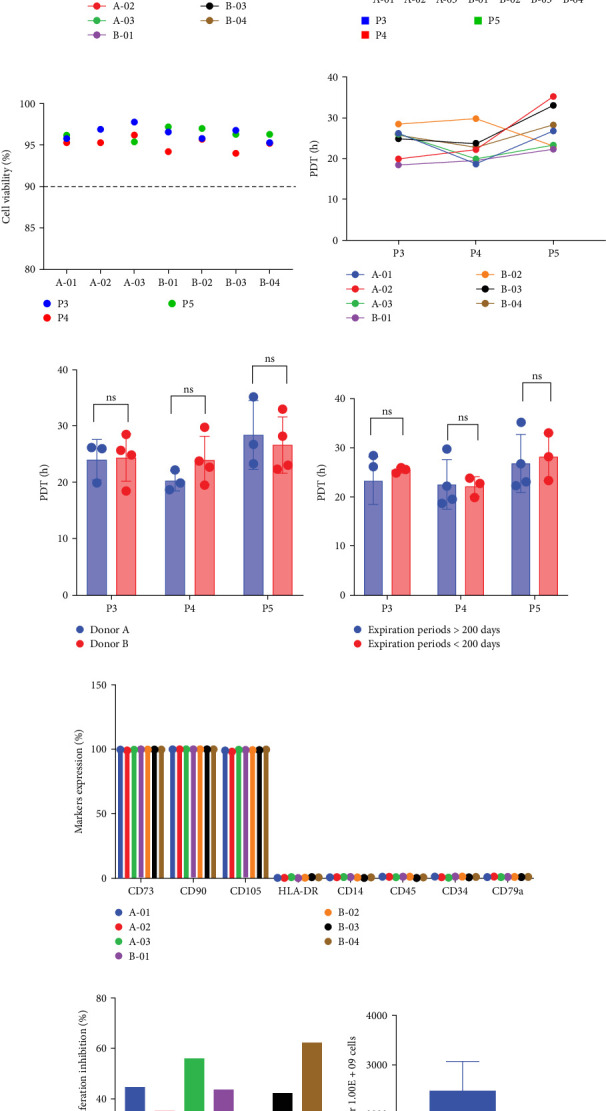
Stability of UC-MSCs production on a large scale in NutriStem XF medium supplemented with 2% HPL. (A–D) Differences in cell number (A), population doubling (PD) (B), cell viability (C), and population doubling time (PDT) (D) among the seven production batches from two donors. (E) Comparison of average PDT in P3, P4, and P5 between batches with donor A versus donor B. (F) Comparison of average PDT in P3, P4, and P5 between batches with longer versus shorter remaining expiration periods of the medium. (G) Expression levels of positive markers (CD105, CD73, and CD90) and negative markers (CD45, CD34, CD14, CD79a, and HLA-DR) among the seven production batches. (H) Lymphocyte proliferation inhibition rates across all batches. (I) The average cost of culture medium per production of 1 × 10^9^ MSCs. ns, not significant.

All batches exhibited robust and consistent expansion capabilities. By P5, the average cell yield reached 9.89 × 10^9^± 1.95 × 10^9^ cells, reflecting a cumulative proliferation fold increase of 4049.90 ± 1498.09 and cumulative PDs of 11.92 ± 0.57 from P2 to P5 (Figure 6A, B and Supporting Information 1: Table S2). Cell viability consistently exceeded 90% across all passages and batches, underscoring the reliability of the medium in supporting high-quality cell production (Figure 6C).

The average PDT of P3, P4, and P5 was 24.21 ± 3.64, 22.34 ± 3.76, and 27.40 ± 5.09 h, respectively (Figure 6D). Although a moderate increase in PDT was observed at P5, the differences were not statistically significant. Similarly, interbatch variations in growth kinetics were noted but did not reach statistical significance. Further analysis indicated that neither the donor source nor the remaining shelf-life of the culture medium had a significant effect on cell proliferation rates (Figure 6E, F). Although not statistically significant, batches using fresher medium showed a trend toward shorter PDT in P3 and P5, suggesting that medium shelf-life may subtly influence performance in prolonged cultures.

Phenotypic characterization confirmed that all batches met ISCT criteria, with positive markers (CD73, CD90, and CD105) expressed in ≥95% of cells and negative markers (CD45, CD34, CD14, CD79a, and HLA-DR) in ≤2% (Figure 6G). Functionally, all batches retained trilineage differentiation potential (osteogenic, adipogenic, and chondrogenic) and exhibited substantial immunomodulatory capacity, with lymphocyte suppression rates exceeding 25% in MLRs (Supporting Information 1: Figure S1; Figure 6H). The culture medium production cost was estimated at $2472.77 ± 599.16 per 1 × 10^9^ cells (Figure 6I). In conclusion, the selected medium system demonstrated scalable, reproducible, and stable expansion of UC-MSCs, fulfilling critical requirements for clinical-grade manufacturing.

## 4. Discussion

hUC-MSCs, which are derived from Wharton's jelly of the UC after fetal birth, exhibit significant in vitro expansion potential and are promising candidates for advanced therapeutic medicinal products [9]. These cells have undergone extensive evaluation in numerous clinical trials [26, 27]. However, discrepancies in clinical trial outcomes have been observed [28], with the preparation of MSCs identified as a key determinant of these outcomes [29]. Various factors, including culture medium, glucose levels, oxygen levels, and seeding density, play crucial roles in influencing the phenotype and functionality of MSCs during in vitro expansion [30]. In particular, several studies have investigated and compared the characteristics of MSCs in different media and supplements, providing valuable insights into the selection of appropriate culture media [21, 31, 32]. In this study, we conducted a comparative analysis of multiple culture systems for UC-MSCs, presenting data on selected media for large-scale manufacturing and providing a comprehensive overview of the entire selection process for GMP manufacturing.

As a critical raw material, the culture systems for hMSCs can be categorized into serum-containing media, serum-free media, xeno-free media, and chemically defined media, with serum-free and xeno-free media emerging as current trends [33]. While FBS was initially utilized for MSC culture, concerns regarding its safety and lot-to-lot variability have hindered its widespread use in GMP manufacturing [34]. In contemporary practice, HPL, which facilitates cell adhesion by providing essential cell attachment factors and is enriched with various growth factors that promote cell proliferation, has emerged as a viable alternative to FBS for hMSC expansion [35, 36, 37, 38].

The Working Party on Cellular Therapies of the International Society of Blood Transfusion has provided compelling evidence supporting the safe and effective use of HPL as a substitute for FBS in the animal serum-free expansion of human cells for clinical transplantation. Moreover, they have proposed recommendations regarding manufacturing and quality management practices [39]. A survey conducted among centers affiliated with the European Society for Blood and Marrow Transplantation revealed that 77% of these centers currently incorporate HPL in their clinical trials [40].

In alignment with these prevailing trends, this study employs GMP-grade HPL, which is compliant with 21 CFR Part 210–211, as a supplement to the culture media. The certificate of analysis for the HPL includes assessments for endotoxin levels, mycoplasma contamination, sterility, pH, osmolality, total protein content, verification of cell growth, and screening for adventitious agents. By adhering to these stringent quality control measures and utilizing GMP-grade HPL, this study ensures the reliability and safety of the culture system for hMSC expansion, thereby enhancing the translational potential of MSC-based therapies.

In addition to the crucial role of serum or its substitution in sustaining and propagating MSC cultures, the selection of a suitable basal medium is equally vital for preserving their characteristics and multipotent properties in vitro [41]. Commonly used basic media for mammalian cell growth include αMEM, DMEM, IMDM, and DMEM/F12 [42]. Sotiropoulou et al. [14] demonstrated that IMDM was inadequate to support BM-MSC culture, while αMEM yielded more cells than did DMEM. Nekanti et al. [43] reported that DMEM-F12 outperformed DMEM in supporting the in vitro expansion of WJ-MSCs. Salehinejad et al. [44] reported that αMEM provided stronger support for UC-MSC growth than did DMEM/F12. In our study comparing three different basic media (αMEM, DMEM, and DMEM/F12) for the in vitro expansion of UC-MSCs, we observed that DMEM exhibited a relatively poor proliferation effect, while αMEM and DMEM/F12 demonstrated superior proliferation effects.

These functional differences may be attributed to variations in the nutrient composition of each medium. Specifically, DMEM contains fewer amino acids compared to αMEM and DMEM/F12, which may limit protein synthesis and impair cellular proliferation. In contrast, DMEM and DMEM/F12 contain higher vitamin concentrations than αMEM, providing better support as coenzymes and prosthetic groups essential for metabolic processes. It is worth noting that a glucose concentration of 1000 mg/L, as used in αMEM and DMEM in this study, has previously been shown to adequately sustain MSC growth and biosynthesis [14]. However, DMEM/F12 is typically formulated with a higher glucose content (3500 mg/L), which may further enhance its nutritional profile. Additionally, although αMEM from Lonza and Gibco are broadly similar, differences in ribonucleoside and deoxyribonucleoside composition—critical for DNA synthesis—could account for minor variations in MSC expansion between the two formulations. Collectively, the simplified composition of DMEM, particularly its reduced array of nonessential amino acids, likely underlies its diminished capacity to support MSC proliferation in our experiments. These insights underscore the importance of medium composition in influencing MSC growth and metabolism.

Furthermore, we conducted comparisons involving different concentrations of platelet lysate (5% or 10% HPL) supplemented in basic media and found little difference between the concentrations, indicating that 5% HPL was adequate to meet the proliferation requirements of MSCs. This finding is consistent with a previous study by Shansky et al. [45] which demonstrated that 5% HPL is superior to 1% HPL, but 10% HPL did not promote more rapid growth of adipose-derived MSCs (AT-MSCs). Additionally, we also compared different brands of HPL (stemulate or PLTGold), revealing minimal differences in their effects on MSC proliferation. This indicates that the specific production methods and composition of the different HPL products had little impact on the proliferative capacity of the MSCs.

Notably, several commercial serum-free and xeno-free mesenchymal stem cell culture media are now available, including StemPro MSC SFM, MesenCult-XF, NutriStem XF, StemMACS MSC expansion media kit XF, PowerStem MSC1, MSCGM MSC growth medium BulletKit, RoosterNourish MSC-XF, Corning MSC Xeno-Free SFM, and PRIME-XV MSC Expansion XSFM [21, 46, 47]. These media have been optimized by formulating basal components and adding purified or recombinant proteins and growth factors, such as human serum albumin, transferrin, insulin, EGF, fibroblast growth factor (FGF), and PDGF, to promote MSC growth. However, they generally lack extracellular matrix proteins to support cell adhesion [48, 49].

In the present study, we focused on three MSC culture media: NutriStem XF, Corning MSC Xeno-Free SFM, and PRIME-XV MSC Expansion XSFM. We found that these media could not solely support the culture of primary cells, and the addition of 2% HPL was required to enable cell growth in all three media. This finding is consistent with the work of Riis et al. [25], who demonstrated that synthetic media were unsuitable for the initiation of adipose-derived stem cell (ASC) cultures from the stromal vascular fraction (SVF) and that culture in HPL was necessary to yield ASCs. This indicates that primary cells require more abundant growth factors and that HPL not only contains rich adhesion factors but also a rich array of growth factors essential for the successful expansion of these primary cell populations.

Few studies have directly compared basic culture media and commercial MSC-specific media for the expansion and maintenance of MSCs. In the present study, we compared the performance of DMEM, DMEM/F12, NutriStem XF, Corning MSC Xeno-Free SFM, and PRIME-XV MSC Expansion XSFM in supporting the growth and functional properties of MSCs.

Overall, the cellular morphology, multilineage differentiation potential, and expression of the CD73, CD90, and CD105 markers (>95%) with low HLA-DR (<2%) under all culture conditions conformed to the minimal criteria for identifying MSCs [5]. However, we found that commercial MSC media had significant advantages over basic media in terms of passage culture, exhibiting lower PDTs, smaller cell diameters, and higher CFU-F. Furthermore, cells cultured in commercial MSC media exhibited a more three-dimensional morphology, suggesting a more favorable cellular state. Additionally, the lymphocyte proliferation suppression rate, an important indicator of MSC immunomodulatory potency, was greater in commercial MSC media than in basic media [50, 51, 52]. These findings are consistent with those reported by other researchers, highlighting the importance of selecting an appropriate culture medium to maintain the relevant biological functions of MSCs [20, 31].

Interestingly, we also observed differences in the expression of CD146 and SSEA-4 in cells cultured under different conditions. The CD146+ MSC subpopulation has been associated with antiaging and stemness properties, enhanced immunosuppressive functions, and greater therapeutic potential [53, 54, 55, 56]. SSEA-4 has also been considered a marker of a stemness subpopulation in adult MSCs [57, 58]. Research by Musiał-Wysocka et al. [59] demonstrated that Wharton's jelly-derived MSCs express pluripotency markers such as NANOG, OCT-4, and SSEA-4, confirming their nontumorigenic behavior in vivo. Our results suggest that different culture media may select and maintain distinct functional MSC subpopulations, which could have important implications for their therapeutic applications.

Genetic stability and tumorigenicity are critical factors in ensuring the biosafety of MSCs for clinical use [60]. During in vitro culture, cells are subjected to an artificial environment and manipulative stress that can increase DNA damage and affect genetic stability [61]. The FDA has suggested that the in vitro expansion of MSCs increases the risk of generating genetically abnormal cells, and monitoring genetic stability should be part of the characterization of cell lines intended for clinical use [62]. Previous studies have provided some insights into the potential risks associated with the use of growth factor cocktails during cell expansion. For example, Al-Masawa et al. [63] reported that a specific growth factor cocktail had a low risk of inducing genotoxic and tumorigenic effects on chondrocytes up to passage 6, with 16.6 PDs. Similarly, other research has shown that growth factors, such as EGF, bFGF, and PDGF-BB, can lead to the activation of oncogenic transcription factors and enhance the tumorigenic potential of MSCs [64, 65]. Therefore, it is essential to evaluate the tumorigenicity and genetic stability of MSCs when assessing different culture media [49].

In the present study, we evaluated the long-term culture of MSCs in selected media using multiple generations of soft agar assays, in vivo tumorigenicity assays, and karyotype analysis to investigate the risk of tumorigenicity. The results showed no evidence of tumorigenic potential in the MSCs cultured in the tested media.

Finally, we scaled up the culture process to 16 10-layer cell factories to verify the stability of MSCs in multibatch production. To the best of our knowledge, this is the first study to evaluate medium stability in large-scale manufacturing for up to seven batches from two donors. In our previous work, we found that the PDT increased as the remaining expiration periods of the media decreased. Therefore, in the large-scale manufacturing process, we controlled the remaining expiration time to be more than 160 days. Ultimately, the PDT remained stable, and the scale-up was successfully completed. From a translational perspective, the estimated medium production cost of approximately $2472 per 1 × 10^9^ cells provides a tangible benchmark for future economic modeling of UC-MSCs therapies.

While this study provides comprehensive insights into the optimization of culture media for UC-MSCs expansion, several limitations should be acknowledged. First, the use of different HPL concentrations across media groups—5% in basal media versus 2% in commercial serum-free formulations—may introduce a potential confounder in direct comparisons. Although this reflects standard supplementation protocols tailored to each system, future studies should aim to equilibrate HPL concentrations across all conditions to more accurately isolate medium-specific effects. Second, large-scale validation was performed only with NutriStem XF medium, selected for its robust immunomodulatory properties. Although this choice was justified based on overall performance, further scale-up studies with other high-performing media, such as Prime-XV, which showed superior proliferation kinetics, would provide a more complete assessment of manufacturing suitability. Finally, the limited number of production batches restricts the generalizability of long-term stability conclusions. The current analysis focused solely on the influence of remaining shelf life on PDT, without examining the effects of storage conditions (e.g., 4 vs. –20°C), duration after opening, or interbatch variability of the culture media on cellular performance. Future investigations involving larger batch numbers will be essential to conclusively demonstrate process robustness and reproducibility required for clinical translation.

## 5. Conclusions

In summary, this study systematically compared the performance of various culture systems for the in vitro expansion of UC-MSCs, providing valuable insights into the influence of culture media on the biological properties and functional heterogeneity of MSCs. Among the tested media, α-MEM and DMEM/F12 exhibited superior proliferative effects compared to those of DMEM. Commercial MSC media, including NutriStem XF, Corning MSC Xeno-Free SFM, and PRIME-XV MSC Expansion XSFM, demonstrated significant advantages in terms of passage culture, cellular morphology, and immunosuppressive potential. The findings confirm that proliferation during primary isolation and passage, adherence to ISCT criteria, functional assays, and tumorigenicity assessments are crucial for selecting an appropriate culture medium. This enhances the translational potential of MSC-based therapies. Further large-scale and multibatch production studies are warranted to ensure the stability and consistency of the MSC culture media.

## Data Availability

The data that support the findings of this study are available from the corresponding author upon reasonable request.

## Supporting Information

Supporting Information Table S1: Comprehensive comparison of various UC-MSC culture media. Table S2: Cell proliferation data for selected media applications in large-scale UC-MSC production. Figure S1: Differentiation potential of seven batches of large-scale manufactured UC-MSCs into osteogenic, adipogenic, and chondrogenic lineages.
