# Supplementary material for: Optimization of Culture Media for Human Umbilical Cord-Derived Mesenchymal Stem Cell Production
Source: Stem Cells Int. 2025 Nov 14;2025:4806605. doi: 10.1155/sci/4806605 (PMC12638159; doi:10.1155/sci/4806605)

**Supplementary Table S1** Comparison of UC-MSCs culture media. “+” indicates higher scores.

|  | α-MEM with 5% hPL | DMEM with 5% hPL | Corning® MSC Xeno-Free SFM with 2% hPL | MSC NutriStem® XF Medium with 2% hPL | Prime-XV MSC Expansion XSFM with 2% hPL |
| --- | --- | --- | --- | --- | --- |
| Proliferative capacity for initial culture | + + | + + | + + | + | + + + |
| Proliferative capacity for passage culture | + | + | + + + | + + + | + + + |
| Cell morphology | + | + | + + | + + | + + |
| In vitro three-lineage differentiation | + + | + + | + + | + + | + + |
| Phenotypic characterization | + + + | + + + | + + + | + + + | + + + |
| Clonogenic potential | + | + | ++ | +++ | +++ |
| Immunomodulatory ability | + | + | + + | + + + | + + |
| GMP compliance | + | + | + + | + + | + + |
| Cost per mL | + + + | + + + | + + | + + | + |
| Supply availability | + + + | + + + | + + | + + + | + + |

**Supplementary Table S2** Cell proliferation data of selected medium application in large-scale production of UC-MSCs

| Donor | Batch | Passage | Seeding density (cells/cm^2^) | Seeding surface area (cm^2^) | Culture duration (hour) | Harvested Cell number | Cell diameter (µm) | Cell viability | Proliferation fold | Population doubling | Population doubling time (h) |
| --- | --- | --- | --- | --- | --- | --- | --- | --- | --- | --- | --- |
| A | 01 | P3 | 5995 | 1×2×623 | 90.83 | 8.51E+07 | 16.72 | 95.80% | 11.08 | 3.48 | 26.17 |
| A | 01 | P4 | 5316 | 5×5×623 | 80.5 | 1.65E+09 | 16.97 | 95.30% | 19.89 | 4.32 | 18.66 |
| A | 01 | P5 | 7956 | 16×10×623 | 101.17 | 1.09E+10 | 18.43 | 96.20% | 13.74 | 3.79 | 26.76 |
| A | 02 | P3 | 5995 | 1×2×623 | 95 | 1.37E+08 | 16.8 | 96.90% | 27.42 | 4.79 | 19.89 |
| A | 02 | P4 | 6874 | 5×5×623 | 87 | 1.62E+09 | 18.31 | 95.30% | 15.17 | 3.93 | 22.18 |
| A | 02 | P5 | 7484 | 16×10×623 | 108.75 | 6.40E+09 | 17.59 | 95.30% | 8.51 | 3.1 | 35.2 |
| A | 03 | P3 | 5995 | 1×2×623 | 91 | 1.00E+08 | 17.38 | 97.80% | 12.61 | 4.4 | 25.98 |
| A | 03 | P4 | 6047 | 5×5×623 | 92.07 | 1.72E+09 | 17.69 | 96.20% | 14.7 | 3.7 | 19.89 |
| A | 03 | P5 | 7937 | 16×10×623 | 112.58 | 8.41E+09 | 18.29 | 95.40% | 10.63 | 3.65 | 23.29 |
| B | 01 | P3 | 4438 | 1×2×623 | 114.5 | 1.59E+08 | 16.96 | 96.60% | 27.03 | 4.77 | 18.46 |
| B | 01 | P4 | 6911 | 5×5×623 | 70 | 1.49E+09 | 17.3 | 94.20% | 12.05 | 3.6 | 19.49 |
| B | 01 | P5 | 8000 | 16×10×623 | 84.75 | 1.12E+10 | 17.62 | 97.20% | 13.99 | 3.82 | 22.27 |
| B | 02 | P3 | 4438 | 1×2×623 | 136.5 | 1.50E+08 | 16.75 | 95.80% | 27.76 | 4.81 | 28.47 |
| B | 02 | P4 | 6899 | 5×5×623 | 117.5 | 1.66E+09 | 16.89 | 95.70% | 15.42 | 3.96 | 29.77 |
| B | 02 | P5 | 7978 | 16×10×623 | 87.67 | 1.11E+10 | 18.01 | 97.00% | 13.95 | 3.81 | 23.05 |
| B | 03 | P3 | 4438 | 1×2×623 | 113.92 | 1.10E+08 | 17.11 | 96.80% | 20.89 | 3.67 | 24.89 |
| B | 03 | P4 | 6526 | 5×5×623 | 73.5 | 1.46E+09 | 17.6 | 94.00% | 12.95 | 3.89 | 23.74 |
| B | 03 | P5 | 7506 | 16×10×623 | 84.83 | 9.35E+09 | 17.73 | 96.30% | 12.49 | 3.42 | 33.01 |
| B | 04 | P3 | 4438 | 1×2×623 | 114.77 | 1.10E+08 | 16.8 | 95.30% | 22.26 | 4.49 | 25.64 |
| B | 04 | P4 | 6945 | 5×5×623 | 93.65 | 2.06E+09 | 16.79 | 95.20% | 17.53 | 4.14 | 22.67 |
| B | 04 | P5 | 7917 | 16×10×623 | 110.54 | 1.19E+10 | 18.05 | 96.30% | 15.13 | 3.93 | 28.2 |

**Supplementary Figure S1** Differentiation potential of UC-MSCs into osteogenic, adipogenic, and chondrogenic lineages


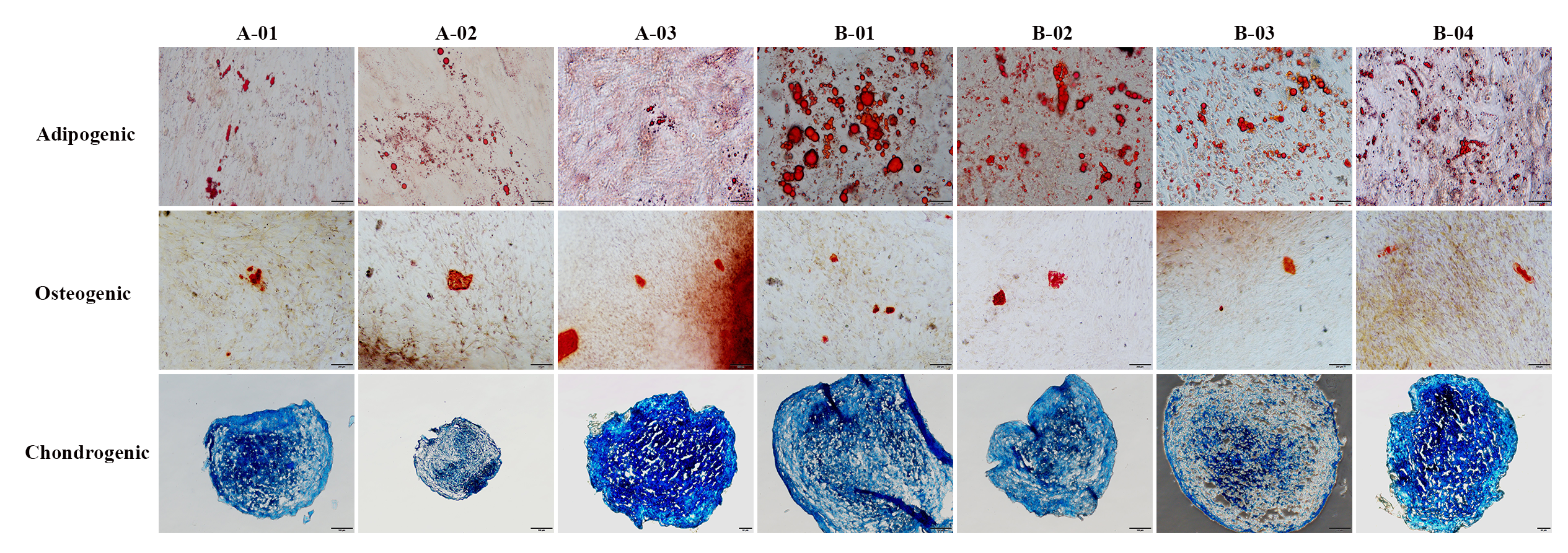

Supplement: Supporting Information — Table S1: Comprehensive comparison of various UC-MSC culture media. Table S2: Cell proliferation data for selected media applications in large-scale UC-MSC production. Figure S1: Differentiation potential of seven batches of large-scale manufactured UC-MSCs into osteogenic, adipogenic, and chondrogenic lineages. [file 4806605.f1.docx]
